# Oral rotavirus vaccine shedding as a marker of mucosal immunity

**DOI:** 10.1038/s41598-021-01288-1

**Published:** 2021-11-05

**Authors:** Benjamin Lee, Md Abdul Kader, E. Ross Colgate, Marya Carmolli, Dorothy M. Dickson, Sean A. Diehl, Masud Alam, Sajia Afreen, Josyf C. Mychaleckyj, Uma Nayak, William A. Petri, Rashidul Haque, Beth D. Kirkpatrick

**Affiliations:** 1 Translational Global Infectious Diseases Research Center, Larner College of Medicine, University of Vermont, 95 Carrigan Drive, Stafford 208, Burlington, VT 05405 USA; 2 Department of Pediatrics, Larner College of Medicine, University of Vermont, Burlington, VT USA; 3 International Centre for Diarrhoeal Disease Research, Dhaka, Bangladesh; 4 Department of Microbiology and Molecular Genetics, Larner College of Medicine, University of Vermont, Burlington, VT USA; 5 Center for Public Health Genomics and Department of Public Health Sciences, University of Virginia, Charlottesville, VA USA; 6 Division of Infectious Diseases and International Health, University of Virginia, Charlottesville, VA USA

**Keywords:** Vaccines, Paediatric research, Gastroenteritis, Rotavirus

## Abstract

Group A rotaviruses (RVA) remain a leading cause of pediatric diarrhea worldwide, in part due to underperformance of currently approved live-attenuated, oral vaccines in low-and-middle income countries. Improved immune correlates of protection (CoP) for existing oral vaccines and novel strategies to evaluate the performance of next-generation vaccines are needed. Use of oral vaccines as challenge agents in controlled human infection models is a potential approach to CoP discovery that remains underexplored. In a live-attenuated, oral rotavirus vaccine (Rotarix, GlaxoSmithKline) efficacy trial conducted among infants in Dhaka, Bangladesh, we explored the potential for the second dose of the two-dose series to be considered a challenge agent through which RVA immunity could be explored, using fecal virus shedding post-dose 2 as a marker of mucosal immunity. Among 180 vaccinated infants who completed the parent study per protocol, the absence of fecal vaccine shedding following the second dose of Rotarix suggested intestinal mucosal immunity generated by the first dose and a decreased risk of RVA diarrhea through 2 years of life (RR 0.616, 95% CI 0.392–0.968). Further development of controlled human infection models for group A rotaviruses, especially in prospective studies with larger sample sizes, may be a promising tool to assess rotavirus vaccine efficacy and CoPs.

## Introduction

Group A rotaviruses (RVA) are a leading worldwide cause of pediatric acute gastroenteritis, responsible for up to 215,000 pediatric deaths yearly^1^. While significant reductions in the burden of pediatric diarrheal disease have occurred since World Health Organization (WHO) pre-qualification of live-attenuated oral rotavirus vaccines in 2008, their relative underperformance in low-and-middle income countries (LMIC) is a major barrier to optimal disease prevention^2^. Next-generation vaccines and/or novel-use strategies incorporating existing vaccines are necessary to advance progress in combating childhood diarrhea. However, efficient and cost-effective evaluation of such interventions is challenging due to lack of specific immune correlates of protection (CoPs) for RVA that are applicable across the full spectrum of oral and parenteral vaccine candidates, leading to ongoing reliance on time- and resource-intensive efficacy studies utilizing clinical endpoints^3^.

Controlled human infection models (CHIMs) offer a rigorous method for evaluating CoPs for many infectious agents^4^. Such models require careful ethical consideration, as well as selection, validation, and testing of a proper challenge agent that can reliably cause infection at a predictable attack rate in susceptible individuals without risk of significant disease. CHIMs are often not ethical to perform in children, but use of approved oral vaccines as challenge agents, as have been used extensively in the poliovirus field^5–10^, shows promise but remains underexplored in the evaluation of mucosal immunity to RVA and rotavirus CoPs.

Globally, the most widely used rotavirus vaccine is Rotarix (GlaxoSmithKline), which consists of live-attenuated, monovalent (G1P[8]) human rotavirus strain RIX4114 administered in a two-dose oral series. Based on serologic response, most vaccine responders will respond to the first dose^11–13^, with the second dose providing “catch-up” response for initial non-responders. Following an oral dose, detection of fecal vaccine shedding indicates gut replication of vaccine-strain virus, which would only occur in the absence of intestinal mucosal immunity sufficient to prevent RVA infection. Conversely, lack of fecal vaccine shedding following a dose might indicate the presence of neutralizing mucosal immunity, an outcome that might not always be reflected in serum antibody responses. Assessment of fecal vaccine shedding following the second dose of Rotarix vaccine could therefore serve as a “natural” CHIM to assess first-dose response, which would apply to most vaccinated children.

To explore these possibilities, we assessed post-vaccination fecal vaccine shedding in PROVIDE, a birth cohort study performed in Dhaka, Bangladesh that included a Rotarix vaccine efficacy trial^14^. We hypothesized that failure to detect vaccine shedding after the second dose, indicating mucosal immunity sufficient to neutralize vaccine-strain virus, would be associated with protection from RVA diarrhea through 2 years of life.

## Results

### Vaccine shedding following Rotarix dose 2

Among 180 evaluable infants, 36 (20%) had fecal shedding of RVA by real-time quantitative reverse transcription polymerase chain reaction (qPCR) detection 3–5 days following the second dose of Rotarix. Among positive specimens, 32 (89%) were confirmed as Rotarix vaccine strain by gene sequencing, 2 (5.5%) were wild-type P[8] strains, and 2 (5.5%) were untypeable due to inability to generate analyzable sequence data. Because vaccinated infants with wild-type infections had also failed to inhibit asymptomatic infection at this time point, we included all positive specimens in analysis, irrespective of strain. Only 9 qPCR-positive specimens (25%) were also positive by stool RVA antigen enzyme immunoassay (EIA), of which 8 were Rotarix vaccine strain and 1 wild-type P[8] strain, indicating very low quantities of viral replication and shedding in this cohort.

### Vaccine shedding, RVA-specific IgA seropositivity, and RVA diarrhea through 2 years of life

52 (29%) infants had at least one episode of EIA-confirmed RVA diarrhea between 18 weeks of life (1 week following completion of the vaccine series) and week 104 (2 years) of life. The median age at diagnosis was 47 weeks [interquartile range (IQR) 33–69]. A smaller proportion of children without fecal RVA shedding following the second Rotarix dose subsequently experienced an episode of RVA diarrhea (26%) compared to children who shed RVA in the stool (39%; RR 0.679, 95% CI 0.415–1.110; Table 1), although this did not reach statistical significance. In sensitivity analysis utilizing a slightly higher qPCR cut-off of cycle threshold (Ct) < 36, a significantly smaller proportion of children without fecal RVA shedding following the second Rotarix dose subsequently experienced an episode of RVA diarrhea (24%) compared to children who shed RVA in the stool (40%; RR 0.616, 95% CI 0.392–0.968; Table 1).

**Table 1:** RVA diarrhea between 18 and 104 weeks in vaccinated infants following Rotarix dose 2.

	RVA diarrhea N (%)	No RVA diarrhea N (%)	RR (95% CI)
**RVA shedding Ct cut-off < 34**			
No shedding	38 (26%)	106 (74%)	0.679 (0.415–1.110)
Shedding	14 (39%)	22 (61%)	–
**RVA shedding Ct cut-off < 36**			
No shedding	31 (24%)	96 (76%)	0.616 (0.392–0.968)
Shedding	21 (40%)	32 (60%)	–
**RVA-IgA**			
Seropositive	7 (18%)	33 (83%)	0.533 (0.261–1.090)
Seronegative	44 (33%)	90 (73%)	–
**Shedding plus RVA-IgA**			
No shedding OR seropositive	39 (27%)	108 (73%)	0.597 (0.362–0.984)
Shedding AND seronegative	12 (44%)	15 (56%)	–
No shedding AND seropositive	5 (16%)	27 (84%)	0.482 (0.208–1.117)
Shedding OR seronegative	46 (32%)	96 (68%)	–

Seropositive: RVA-IgA ≥ 20 U/mL; seronegative: RVA-IgA < 20 U/mL.

*CI* confidence interval, *Ct* cycle threshold, *RR* relative risk, *RVA* group A rotavirus.

Next, we compared post-dose 2 shedding to the most widely used measure of oral rotavirus vaccine immunogenicity, total RVA-specific IgA (RVA-IgA). Among 174 infants with both shedding and RVA-IgA data available for analysis, RVA-IgA seropositivity was associated with a reduction in risk of RVA diarrhea through 2 years of life (18% vs. 33%; RR 0.533, 95% CI 0.261–1.090; Table 1) that was generally similar in magnitude to that observed in children without post-dose 2 shedding, although this did not reach statistical significance. There was no association between plasma RVA-IgA seropositivity and post-dose 2 shedding: 23% of infants who shed vaccine were RVA-IgA seropositive (N = 8/35), which was identical to the proportion of infants who did not shed vaccine who were RVA-IgA seropositive (N = 32/139; *P* = 1.0). When combining both measures together, children who either inhibited fecal RVA shedding following the second Rotarix dose or were RVA-IgA seropositive had decreased risk for RVA diarrhea through 2 years of life (27% vs. 44%; RR 0.597, 95% CI 0.362–0.984; Table 1). When comparing RVA diarrhea in children who both inhibited fecal RVA shedding AND were RVA-IgA seropositive, which would presumably identify children with the strongest evidence for RVA immunity, to those who either only shed RVA or were RVA-IgA seropositive, results were generally similar to those assessing RVA-IgA seropositivity alone (16% vs. 32%; RR 0.482, 95% 0.208–1.117; Table 1), although results did not reach statistical significance. All additional children identified with RVA shedding using the higher cutoff of Ct < 36 in sensitivity analysis were RVA-IgA seropositive, therefore these results for the combined outcomes remained unchanged.

**Figure 1:**
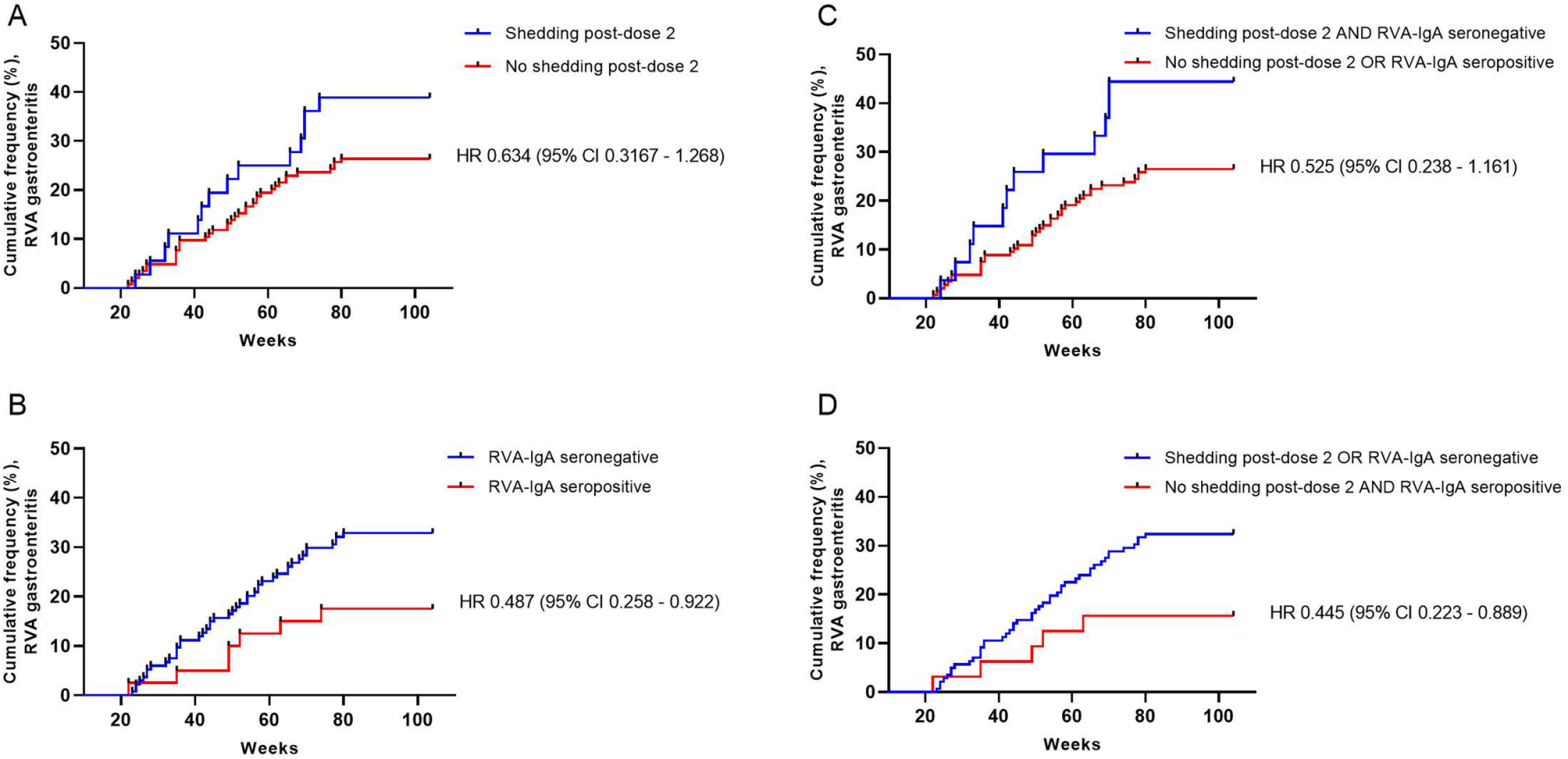
Time to first episode of RVA diarrhea among vaccinated infants 18–104 weeks of life. (**A**) Kaplan–Meier curve of time to first episode of RVA diarrhea in children with (blue line) or without (red line) fecal RVA shedding following Rotarix dose 2. (**B**) Kaplan–Meier curve of time to first episode of RVA diarrhea in children who were RVA-IgA seronegative (< 20 U/mL, blue line) or seropositive (≥ 20 U/mL, red line) at week 18 of life. (**C**) Kaplan–Meier curve of time to first episode of RVA diarrhea in children with both fecal RVA shedding following Rotarix dose 2 and who were RVA-IgA seronegative (blue line) or children who either inhibited fecal RVA shedding following Rotarix dose 2 or were RVA-IgA seropositive (red line). (**D**) Kaplan–Meier curve of time to first episode of RVA diarrhea in children with fecal RVA shedding following Rotarix dose 2 or who were RVA-IgA seronegative (blue line) or children who both inhibited fecal RVA shedding following Rotarix dose 2 and were RVA-IgA seropositive (red line). *CI* confidence interval, *HR* hazard ratio. Kaplan–Meier estimators followed by log-rank test was performed using GraphPad Prism version 9.2.0 for Windows (GraphPad Software, San Diego, CA, USA; https://www.graphpad.com/).

Children who inhibited fecal RVA shedding post-dose 2 qualitatively appeared to have slightly increased time to the first episode of RVA diarrhea compared to those who shed virus post-dose 2 (HR 0.634, 95% CI 0.317–1.268; Fig. 1A), although this did not reach statistical significance. Similarly, children who were RVA-IgA seropositive at 18 weeks had increased time to first episode of RVA diarrhea compared to seronegative children (HR 0.487, 95% CI 0.258–0.922; Fig. 1B). When both measures were combined, those who inhibited fecal shedding post-dose 2 OR were RVA-IgA seropositive had a slightly increased time to the first episode of RVA diarrhea compared to those were both RVA-IgA seronegative and shed virus post-dose 2 (HR 0.525, 95% CI 0.238–1.161; Fig. 1C), although this did not reach statistical significance. Children who inhibited fecal shedding post-dose 2 AND were RVA-IgA seropositive had an increased time to the first episode of RVA diarrhea compared to those who were either RVA-IgA seronegative or shed virus post-dose 2 (HR 0.445, 95% CI 0.223–0.889; Fig. 1D).

## Discussion 

As assessed among young children in Dhaka, Bangladesh, lack of RVA shedding following the second dose of Rotarix appeared to be associated with protection from subsequent RVA diarrhea through 2 years of life, although outcomes of some analyses did not reach strict statistical significance. To our knowledge, these are the first data that evaluate vaccine shedding in the context of a CHIM coupled with RVA diarrhea incidence and not simply as a measure of potential vaccine take. Immunity from RVA gastroenteritis is unlikely to require sterilizing immunity, meaning that protection from diarrhea may occur despite asymptomatic or mild infection^16–18^. However, our results suggest that protection from asymptomatic infection by vaccine-strain virus in a human challenge model may yet be an informative surrogate for clinical protection from RVA gastroenteritis. While the directions of the effects observed in our dataset were clear, the precision of our estimates was low due to limitations in sample size, as evidenced by our large confidence intervals, which in some analyses included 1 (indicating failure to meet strict statistical significance), and by the timing of sample collection. Therefore, these data require further confirmation in larger prospective studies. Nevertheless, the results suggest that CHIMs utilizing approved oral rotavirus vaccines warrant further development, particularly in contexts where the standard immunogenicity measure RVA-IgA may not be an appropriate outcome, such as with next-generation, non-replicating vaccines^19^.

Lack of association between post-dose 2 shedding and plasma RVA-IgA seropositivity suggests that lack of shedding may detect mucosal immunity that may not be always be reflected in plasma/serum antibody measurement. Nevertheless, inhibition of fecal shedding in our model did not appear to offer any appreciable advantage over RVA-IgA, which is currently the best available CoP for RVA, despite its limitations^3,20^. However, the RVA-IgA assay predominantly measures antibodies against the RVA middle capsid structural protein VP6, which is the immunodominant antigen in the humoral response to RVA. VP6 is available for recognition only after successful infection of host intestinal cells leads to uncoating of the outer capsid layer to reveal VP6 and initiate viral replication^21^. Therefore, alternate assays are likely needed to adequately assess immune responses for next-generation, non-replicating vaccines that use antigens other than RVA VP6^19^. Rotarix shedding has been used in phase 1 and 2 trials of a P2-VP8* parenteral vaccine candidate to evaluate for induction of mucosal immunity^22,23^. In a phase 1/2 safety and immunogenicity trial of the trivalent P2-VP8* formulation, infants who received the highest dose of 90 ug had significantly reduced rates of PCR-confirmed Rotarix shedding following the first Rotarix dose compared to infants who received placebo^22^. A phase 3 efficacy trial of this vaccine is currently in progress (Clinicaltrials.gov NCT04010448), but no data currently exist regarding vaccine shedding and vaccine efficacy for parenteral vaccines.

Our results likely underestimated the potential impact of inhibition of vaccine shedding, since we were only able to assess vaccine shedding following the second dose of the vaccine, and not at a subsequent time point after completion of the full vaccine series. Based on RVA-IgA immunogenicity data, early studies from Latin America and Finland suggest that anywhere from 8 to 23% of vaccinated infants did not respond to the first dose of vaccine but responded to the second (our calculation)^12,13^. Thus, in this study a subset of children who did not respond to the first dose of Rotarix may have responded to the second dose and shed vaccine. If so, they may have acquired immunity to future infection due to the second dose of vaccine and may have inhibited RVA shedding if challenged with an additional dose at a later time point, meaning such children would have been misclassified here, even if the total number likely comprised a relatively small proportion of participants. Another factor that may have led to underestimate of immune response is measurement of antibody response 1 week after the second dose, which would have reflected first-dose response, but may have been too early to capture second-dose immune responses, as explained in further detail elsewhere^20^.

It is also possible that inhibition of post-dose 2 shedding was due to factors other than a response to the first vaccine dose. For example, high levels of maternally derived, RVA-specific serum IgG antibodies are associated with decreased oral rotavirus vaccine immunogenicity^20,24,25^. Infants who inhibited vaccine shedding due to high maternal antibodies may have become susceptible to RVA infection later in life as these antibodies waned in the absence of an adequate vaccine response to replace them. Unfortunately, only a subset of infants in our parent study had pre-vaccination RVA-specific IgG (RVA-IgG) antibodies measured, such that only 59 infants had both post-dose 2 shedding and pre-vaccination RVA-IgG data available for analysis; this small sample size precluded meaningful analysis.

We previously attempted to consider dose 1 response by evaluation of post-dose 1 shedding, and reported that post-dose 1 shedding as a marker for successful vaccine take or post-dose 1 shedding followed by lack of shedding post-dose 2 was not associated with subsequent year 1 RVA diarrhea^19,26^. However, among infants with post-dose 1 shedding specimens available for analysis (N = 176), 73% had specimens collected within 2 days following vaccination, meaning many detections at this time point could reflect passage of vaccine inoculum and not shedding indicative of viral replication, confounding interpretation of results relying on post-dose 1 shedding as a marker for successful vaccine take. Similarly, the possibility that detection of shedding post-dose 2 in some children could merely indicate passage of vaccine inoculum, rather than vaccine replication, cannot be completely ruled out.

In summary, infants in Dhaka, Bangladesh who inhibited fecal shedding of Rotarix, a live-attenuated, oral vaccine, following the second vaccine dose demonstrated a clear trend towards reduced risk for subsequent RVA diarrhea through 2 years of life. Based on these data, further development of novel CHIMS using approved oral rotavirus vaccines as challenge agents to explore immune COPs for RVA appears warranted, particularly for the evaluation of next-generation, non-replicating rotavirus vaccines.

## Methods

### Study population

We conducted a post-hoc exploratory analysis in a subset of participants in the per-protocol, Rotarix-vaccinated arm of PROVIDE (N = 292) in whom a series of stool specimens had previously been collected and stored. PROVIDE was a birth cohort study conducted in the urban Mirpur district of Dhaka, Bangladesh from 2011 to 2014 and included an oral Rotarix vaccine efficacy trial. Full study details have been published^10,14,27^. Briefly, infants were enrolled in the first week of life and randomized 1:1 to receive Rotarix (N = 350) or not receive Rotarix (N = 350) at weeks 10 and 17 of life, with active community diarrheal surveillance through 2 years of age. RVA diarrhea was diagnosed by stool RVA antigen EIA (Oxoid, Hampshire, UK). Vaccine immunogenicity was assessed by measurement of total plasma RVA-specific IgA (RVA-IgA) by EIA. Seropositivity was defined as RVA-IgA > 20 U/mL at week 18 of life, 1 week following the second vaccine dose^20^. The study was approved by the ethical review boards of the University of Vermont, University of Virginia, and the International Centre for Diarrhoeal Disease Research, Bangladesh (icddr,b) and was registered at ClinicalTrials.gov (NCT01375647). Written informed consent for all participants was obtained from a parent. The trial was conducted in accordance with all relevant regulations and guidelines, including the Declaration of Helsinki and International Council on Harmonization Good Clinical Practice Guidelines.

Shortly following study initiation, the protocol was amended to begin collection of an asymptomatic surveillance stool specimen collected between 1 and 3 days after the first Rotarix dose and another 3–5 days after the second Rotarix dose in all infants in the vaccine arm who had not yet been vaccinated. Because Rotarix shedding peaks between 3 and 7 days after vaccination^15^, with earlier detection potentially reflecting passage of the vaccine inoculum through the gastrointestinal tract, we focused on the post-dose 2 specimens. Infants with RVA diarrhea prior to week 18 of life were excluded, leaving 180 vaccinated children with evaluable specimens for study inclusion.

### Laboratory procedures

Surveillance stool specimens underwent total nucleic acid extraction and quantitative real-time reverse transcription polymerase chain reaction (qPCR) for stool RVA detection as previously described^28–30^. Briefly, total nucleic extractions were performed on stool using the QIAamp stool DNA mini kit (QIAGEN, Valencia, CA) followed by qPCR using the CFX96 platform (Bio-Rad, Hercules, CA) using Ultraplex 1-step ToughMix enzyme (Quantabio, Beverly, MA) and previously described primers and probes targeting the NSP3 gene segment of RVA^30^. Specimens were considered positive at qPCR cut-off of Ct < 34, the analytic limit of detection of the assay^15^. We also performed a sensitivity analysis using a cut-off Ct < 36, as we anticipated that many specimens would shed very low quantities of virus. Positive specimens underwent conventional reverse transcription PCR to amplify the RVA VP8* gene segment using QIAGEN OneStep RT-PCR enzyme with VP4F and VP4R primers^31^, followed by Sanger sequencing using VP4F or VP4R primers and BLAST analysis to confirm vaccine versus wild-type RVA, as previously described^28^. All qPCR-positive specimens were additionally tested by stool EIA.

### Statistical analysis

Categorical outcomes were assessed using Chi-square or Fisher’s exact test, as appropriate, to estimate proportion differences. Relative risk (RR) with associated 95% confidence intervals (CI) was calculated from the relevant 2 × 2 tables according to standard methods. Kaplan–Meier estimators were used to calculate cumulative incidence of the first episode of RVA diarrhea through year 2 of life according to RVA-IgA and post-dose 2 shedding status, with differences between groups analyzed using log-rank test. All analyses were performed using SPSS version 27.0.0.0 (IBM, Armonk, NY, USA; https://www.ibm.com/products/spss-statistics) or GraphPad Prism version 9.2.0 for Windows (GraphPad Software, San Diego, CA, USA; https://www.graphpad.com/). Differences were considered statistically significant at two-sided *P* value < 0.05, or when the 95% confidence interval for relative risk or hazard ratio did not include 1.0.

## Data Availability

All data generated during and/or analysed during the current study are available from the corresponding author on reasonable request.
